# Glucocorticoid-mediated ER-mitochondria contacts reduce AMPA receptor and mitochondria trafficking into cell terminus via microtubule destabilization

**DOI:** 10.1038/s41419-018-1172-y

**Published:** 2018-11-14

**Authors:** Gee Euhn Choi, Ji Young Oh, Hyun Jik Lee, Chang Woo Chae, Jun Sung Kim, Young Hyun Jung, Ho Jae Han

**Affiliations:** 1 Department of Veterinary Physiology, College of Veterinary Medicine, Research Institute for Veterinary Science, and BK21 PLUS Program for Creative Veterinary Science Research, Seoul National University, Seoul, 08826 Republic of Korea; 2 Department of Agricultural Biotechnology, Animal Biotechnology Major, and Research Institute of Agriculture and Life Science, Seoul National University, Seoul, 08826 Republic of Korea

## Abstract

Glucocorticoid, a major risk factor of Alzheimer’s disease (AD), is widely known to promote microtubule dysfunction recognized as the early pathological feature that culminates in memory deficits. However, the exact glucocorticoid receptor (GR)-mediated mechanism of how glucocorticoid triggers microtubule destabilization and following intracellular transport deficits remains elusive. Therefore, we investigated the effect of glucocorticoid on microtubule instability and cognitive impairment using male ICR mice and human neuroblastoma SH-SY5Y cells. The mice group that was exposed to corticosteroid, the major glucocorticoid form of rodents, showed reduced trafficking of α-amino-3-hydroxy-5-methyl-4-isoxazole propionic acid receptor (AMPAR) 1/2 and mitochondria, which are necessary for memory establishment, into the synapse due to microtubule destabilization. In SH-SY5Y cells, cortisol, the major glucocorticoid form of humans, also decreased microtubule stability represented by reduced acetylated α-tubulin to tyrosinated α-tubulin ratio (A/T ratio), depending on the mitochondria GR-mediated pathway. Cortisol translocated the Hsp70-bound GR into mitochondria which thereafter promoted GR-Bcl-2 interaction. Increased ER-mitochondria connectivity via GR-Bcl-2 coupling led to mitochondrial Ca^2+^ influx, which triggered mTOR activation. Subsequent autophagy inhibition by mTOR phosphorylation increased SCG10 protein levels via reducing ubiquitination of SCG10, eventually inducing microtubule destabilization. Thus, failure of trafficking AMPAR1/2 and mitochondria into the cell terminus occurred by kinesin-1 detachment from microtubules, which is responsible for transporting organelles towards periphery. However, the mice exposed to pretreatment of microtubule stabilizer paclitaxel showed the restored translocation of AMPAR1/2 or mitochondria into synapses and improved memory function compared to corticosterone-treated mice. In conclusion, glucocorticoid enhances ER-mitochondria coupling which evokes elevated SCG10 and microtubule destabilization dependent on mitochondrial GR. This eventually leads to memory impairment through failure of AMPAR1/2 or mitochondria transport into cell periphery.

## Introduction

Microtubule takes a pivotal role acting as major highway for intracellular trafficking of necessary components such as proteins or organelles. Notably, maintaining homeostasis in microtubule networks in neuronal cells is particularly important for strengthening synaptic connection and regulating axonal transport. Therefore, it is not surprising that microtubule dysfunction and following synaptic transport deficits are commonly observed in neurodegenerative diseases. For instances, reduced microtubule numbers and altered post-translational modification (PTM) of α-tubulins are observed in AD^1^. Microtubule networks are important for consolidating memory via promoting AMPAR translocation into synapse. Previous research already demonstrated that stable microtubule structures promoted AMPAR endocytosis via MAP1B synthesis or the kinesin-1-mediated AMPAR transport, which enhance cognitive function^2,3^. Stable acetylated α-tubulin is also responsible for transporting mitochondria into neuronal cell periphery to provide energy for synaptic homeostasis and memory formation^4^. Thus, microtubule dysfunction precedes memory impairment since neuronal cells failed to import AMPAR and mitochondria into synapses, both of which are necessary to trigger long term potentiation and eventual memory formation. However, even though microtubule dysfunction represents a downstream of neurodegenerative cascades, the mechanism concerning pathogenesis of microtubule destabilization and memory impairment needs further investigation for discovering potential therapeutics of AD.

Stress, a major etiology of AD, is generally believed to induce alterations in microtubule networks through the glucocorticoid signaling pathway. Numerous reports have previously focused on the effect of glucocorticoid on hyperphosphorylation of tau as a key regulator of microtubule destabilization in AD^5^. Recently, however, many changes in microtubule networks have been observed like change in the ratio of acetylated/tyrosinated α-tubulins rather than tau pathology in AD. Namely, it is important to define the detailed mechanisms of glucocorticoid on microtubule dysfunction rather than neurofibrillary tangle formations to find the new neurodegenerative cascades of AD. Glucocorticoid mediates microtubule destabilization via various signaling methods. Growing evidence demonstrates that excessive glucocorticoid inhibited microtubule assembly through activating genomic pathway in rat C_6_ glioma cells^6^ or hyper-stabilizing the tubulin through nongenomic mechanism^7^. However, understanding of how glucocorticoid enhances microtubule dysfunction in neuronal cells and subsequent memory deficits remains unclear. Among the various effects, mitochondrial GR is of interest in the AD pathogenesis since it plays a crucial role in Ca^2+^ homeostasis in mitochondria through interacting with Bcl-2. Aberrant changes of Ca^2+^ in mitochondria can damage the microtubule dynamics through elevating cytoskeletal protein calpains and forming tangles, eventually leading to memory deficits^8^. Thus, identifying how glucocorticoid promotes microtubule dysfunction and memory impairment via changing Ca^2+^ homeostasis is important for understanding molecular links between stress and AD.

In the present study, we used male ICR mice exposed to glucocorticoid to assess how glucocorticoid can affect memory formation. Mice with short-term glucocorticoid treatment during several hours were used to confirm the newly revealed mechanism of mitochondrial Ca^2+^ influx. The mechanisms of microtubule destabilization and following memory deficits were observed in mice underwent relatively longer term of glucocorticoid treatment for 2–3 days. In addition, human neuroblastoma SH-SY5Y cells, widely used as neurodegenerative disease model, were utilized to investigate the detailed mechanism of microtubule dysfunction via GR-mediated changes in mitochondrial Ca^2+^ homeostasis. Overall, we determined detrimental effects of glucocorticoid on microtubule networks followed by memory impairment and the underlying mechanisms using both in vivo and in vitro models.

## Results

### The effect of corticosterone on memory impairment in vivo

**Fig. 1:**
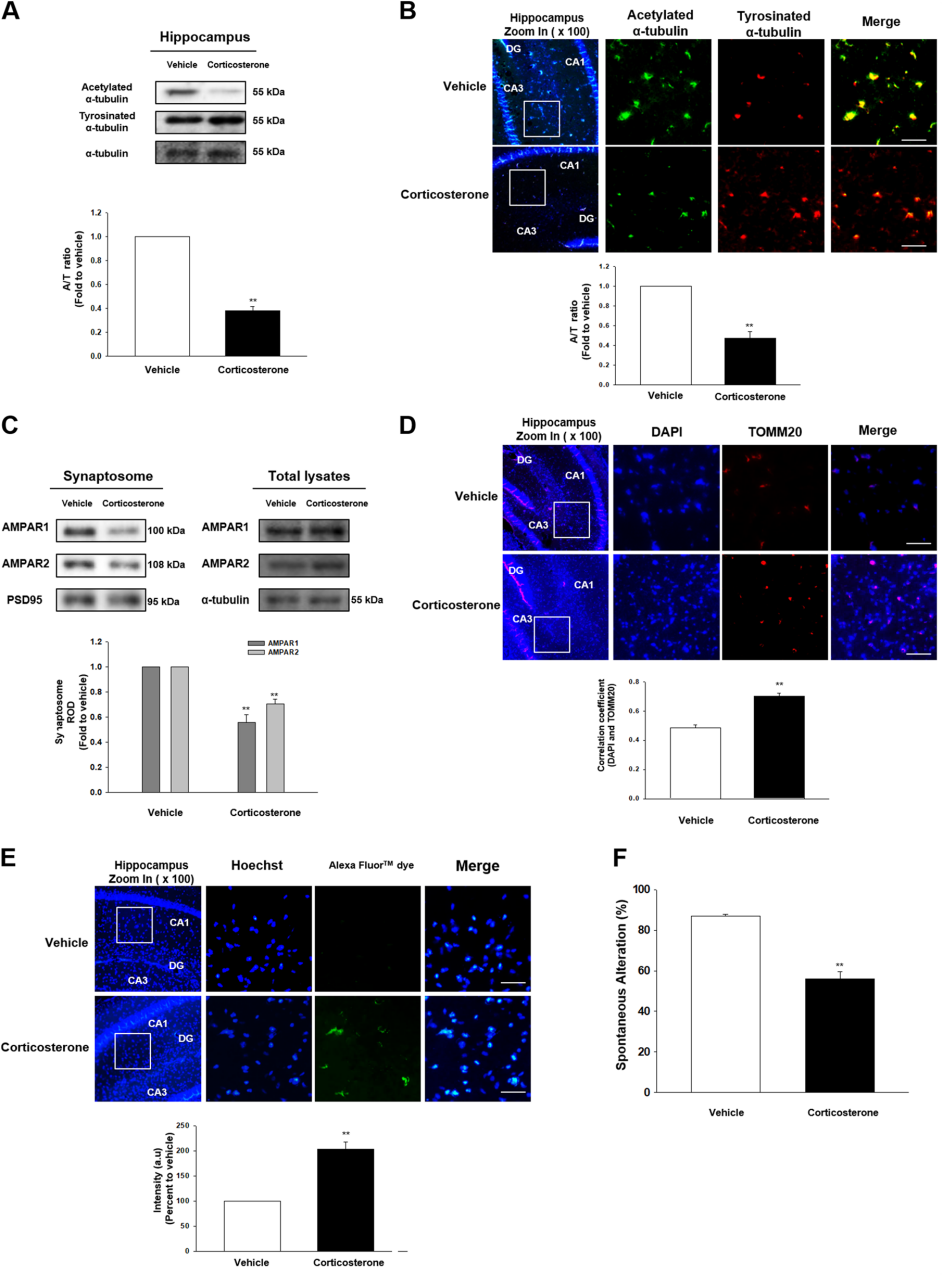
The effect of corticosterone on microtubule destabilization in the male ICR mice. **a** The hippocampus of male ICR mice exposed to vehicle or corticosterone (10 mg/kg) was collected. Acetylated α-tubulin, tyrosinated α-tubulin, and α-tubulin were detected by western blot. ^**^ indicates *p* < 0.01 vs. vehicle. *n* = 5. **b** Slide samples for immunohistochemistry (IHC) were immunostained with acetylated α-tubulin (green), tyrosinated α-tubulin (red), and DAPI (blue). Scale bars, 200 μm (magnification, ×200). ^**^ indicates *p* < 0.01 vs. vehicle. *n* = 5. **c** The synaptic protein was extracted from the hippocampus of mice treated with vehicle or corticosterone (10 mg/kg). Synaptic protein expressions were normalized by loading control of synaptosome, PSD95. Total lysates of hippocampus were also shown in the right panel. ^**^ indicates *p* < 0.01 vs. vehicle. *n* = 5. **d** Slide samples for IHC were immunostained with DAPI (blue) and TOMM20 (red). Scale bars, 200 μm (magnification, ×200). Correlation coefficient analysis using Pearson’s coefficient value between DAPI and TOMM20 was done. ^**^ indicates *p* < 0.01 vs. vehicle. *n* = 5. **e** TUNEL assay was performed using slide samples of hippocampus from mice with vehicle or corticosterone (10 mg/kg). The intensity of green fluorescence indicates the amount of neuronal cell death. Scale bars, 200 μm (magnification, ×200). ^**^ indicates *p* < 0.01 vs. vehicle. *n* = 5. **f** The mice exposed to vehicle or corticosterone (10 mg/kg) were subjected to Y-maze test to evaluate memory function. ^**^ indicates *p* < 0.01 vs. vehicle. *n* = 6. All blot and immunofluorescence images are representative. Quantative data are presented as a mean ± S.E.M.

We first examined microtubule dynamics in hippocampus of male ICR mice treated with corticosterone, the major glucocorticoid form in rodents. Microtubule dynamics can be controlled by the intrinsic GTPase activity of tubulins and various PTMs that occur on C-terminal tails, interacting with motor proteins and microtubule-associated proteins. Acetylated or detyrosinated α-tubulin is the marker of stable tubulin which reduces microtubule depolymerization. In contrast, tyrosinated α-tubulin is the labile tubulin and susceptible to microtubule destabilizing molecules. Thus, A/T ratio has been used to evaluate microtubule stability^9^. Both immunoblotting and immunofluorescent results revealed that corticosterone reduced A/T ratio in hippocampus (Figs. 1a, b). Microtubule destabilization usually exerts decreased transport of necessary components into cell periphery. Thus, a failure to traffick AMPAR1/2, the major subunits for regulating the memory function in hippocampus, into synapse was shown while there were no detectable changes in AMPAR1/2 expressions in mice group with corticosterone (Fig. 1c). In addition, the perinuclear clumping of mitochondria was observed in mice with corticosterone (Fig. 1d), which is the representative phenomenon for microtubule dysfunction. Since mitochondrial trafficking to cell terminus was impaired, cell death would likely follow. We identified that the cell apoptosis was increased with corticosterone treatment using the TUNEL assay (Fig. 1e). With decreased AMPAR and mitochondria transport, the spontaneous alteration percentage for evaluating cognitive function was decreased in corticosterone-treated mice (Fig. 1f). Thus, the results suggest that corticosterone triggered reduced A/T ratio and transport deficits, followed by memory deficits.

### Translocation of GR into mitochondria induced ER-mitochondria tethering

**Fig. 2:**
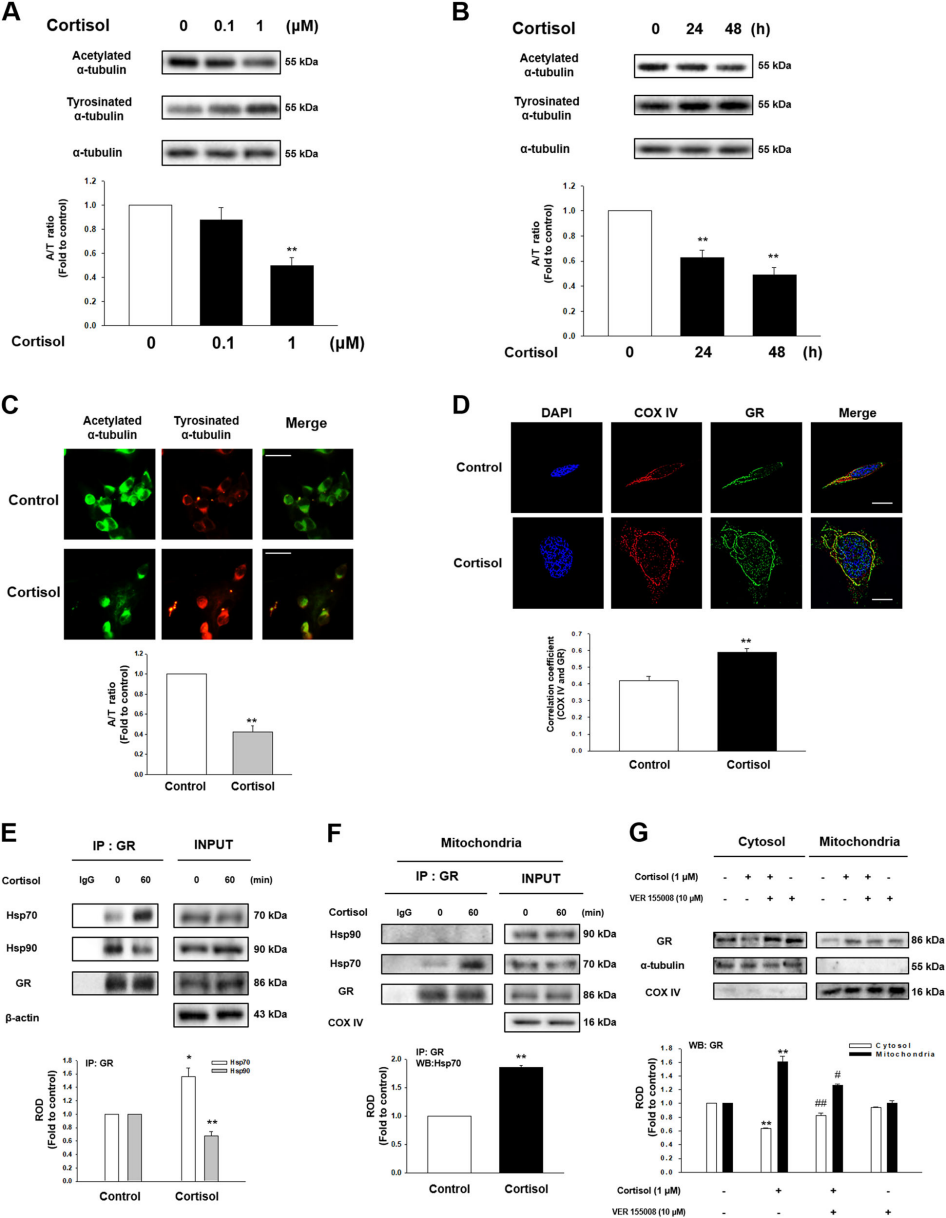
The effect of cortisol on microtubule dysfunction via GR trafficking into mitochondria in SH-SY5Y. **a** Cortisol (0–1 μM) was treated for 48 h in SH-SY5Y cells. Then, acetylated α-tubulin, tyrosinated α-tubulin, and α-tubulin were detected by western blot. ^**^ indicates *p* < 0.01 vs. control. *n* = 5. **b** Total cell lysates in a time response with 1 μM cortisol were subjected to western blot. Acetylated α-tubulin, tyrosinated α-tubulin, and α-tubulin were detected. ^**^ indicates *p* < 0.01 vs. control. *n* = 5. (**c**) Immunostaining of cells treated with cortisol for 48 h were visualized by Eclipse Ts2™ fluorescence microscopy. The green indicates acetylated α-tubulin and the red indicates tyrosinated α-tubulin. Scale bars, 100 μm (magnification, ×400). ^**^ indicates *p* < 0.01 vs. control. *n* = 5. **d** The cells were treated with cortisol (1 μM) for 2 h which were immnunostained with DAPI (blue), COX IV (red) and GR (green). The images were acquired by SRRF imaging system. ^**^ indicates *p* < 0.01 vs. control. Scale bars, 20 μm (magnification, ×1000). *n* = 5. (**e**) The cells were incubated with cortisol (1 μM) for 60 min. GR was co-immunoprecipitated with anti-Hsp70 and -Hsp90 antibodies (the left side). Expression of Hsp70, Hsp90, GR, and β-actin in total cell lysates is shown in the right side. ^*, **^ indicates *p* < 0.05*, p* < 0.01 vs. control, respectively. *n* = 4. **f** The mitochondrial parts of the cells treated with cortisol (1 μM) for 60 min underwent immunoprecipiatation with GR. ^**^ indicates *p* < 0.01 vs. control. *n* = 5. (**g**) The cells were incubated with VER 155008 (10 μM) for 30 min before cortisol treatment (1 μM) for 2 h. Cytosolic and mitochondrial protein expressions were normalized by α-tubulin and COX IV, respectively. ^**^ indicates *p* < 0.01 vs. control. ^#^, ^##^ indicates *p* < 0.05*, p* < 0.01 vs. cortisol, respectively. *n* = 4. All blot and immunofluorescence images are representative. Quantative data are presented as a mean ± S.E.M.

We evaluated the effect of cortisol, the major form of glucocorticoid in humans, on microtubule destabilization in SH-SY5Y cell lines based on in vivo experiments. We showed that cortisol decreased the A/T ratio of cells in a concentration and time dependent manner (Figs. 2a, b). Reduced A/T ratio was also detected in the immunostaining results upon cortisol (Fig. 2c). Cortisol can trigger various signaling including genomic, nongenomic, and mitochondria GR-mediated pathway. To differentiate which signaling induces the change in microtubule dynamics, actinomycin D (nuclear transcription inhibitor) that blocks the genomic pathway of cortisol and the cortisol-BSA, which specifically induces the membrane GR-mediated pathway, were treated. We found that A/T ratio was not recovered in cortisol-treated cells with actinomycin D and no detectable change in the A/T ratio was observed in cortisol-BSA-treated cells (Figs. S1A–S1B). In contrast, mitochondrial GR played an important role in regulation of microtubule dynamics. Co-localization of GR and COX IV, the mitochondrial marker, increased upon cortisol for 2 h (Fig. 2d). Subcellular fraction results also indicated that cortisol induced GR translocation into mitochondria, which was inhibited by RU 486 (competitive inhibitor of GR, Fig. S1C). The inactive form of GR binds to many chaperone proteins. In contrast, the active form of GR detaches from heat shock protein90 (Hsp90) with ligand and is subsequently translocated into nucleus or mitochondria. Some GRs do not detach from Hsp70 or selectively associate with un-bound cytosolic Hsp70; all of which guide the GR to translocate into mitochondria^10^. Consistent with previous research, cortisol promoted GR-Hsp70 coupling while GR-Hsp90 interaction was reduced (Fig. 2e). The immunoprecipitation result of mitochondrial parts also revealed that GR-Hsp70 binding was elevated when exposed to cortisol while there was no GR-Hsp90 complex detected (Fig. 2f). The tendency of increased GR translocation into mitochondria was inhibited with VER 155008 (Hsp70 inhibitor) pretreatment (Fig. 2g). We also found that the reduced A/T ratio was recovered with VER 155008 pretreatment (Fig. S1D), suggesting that mitochondrial GR signaling among the various pathways of glucocorticoid is likely to alter microtubule dynamics.

**Fig. 3:**
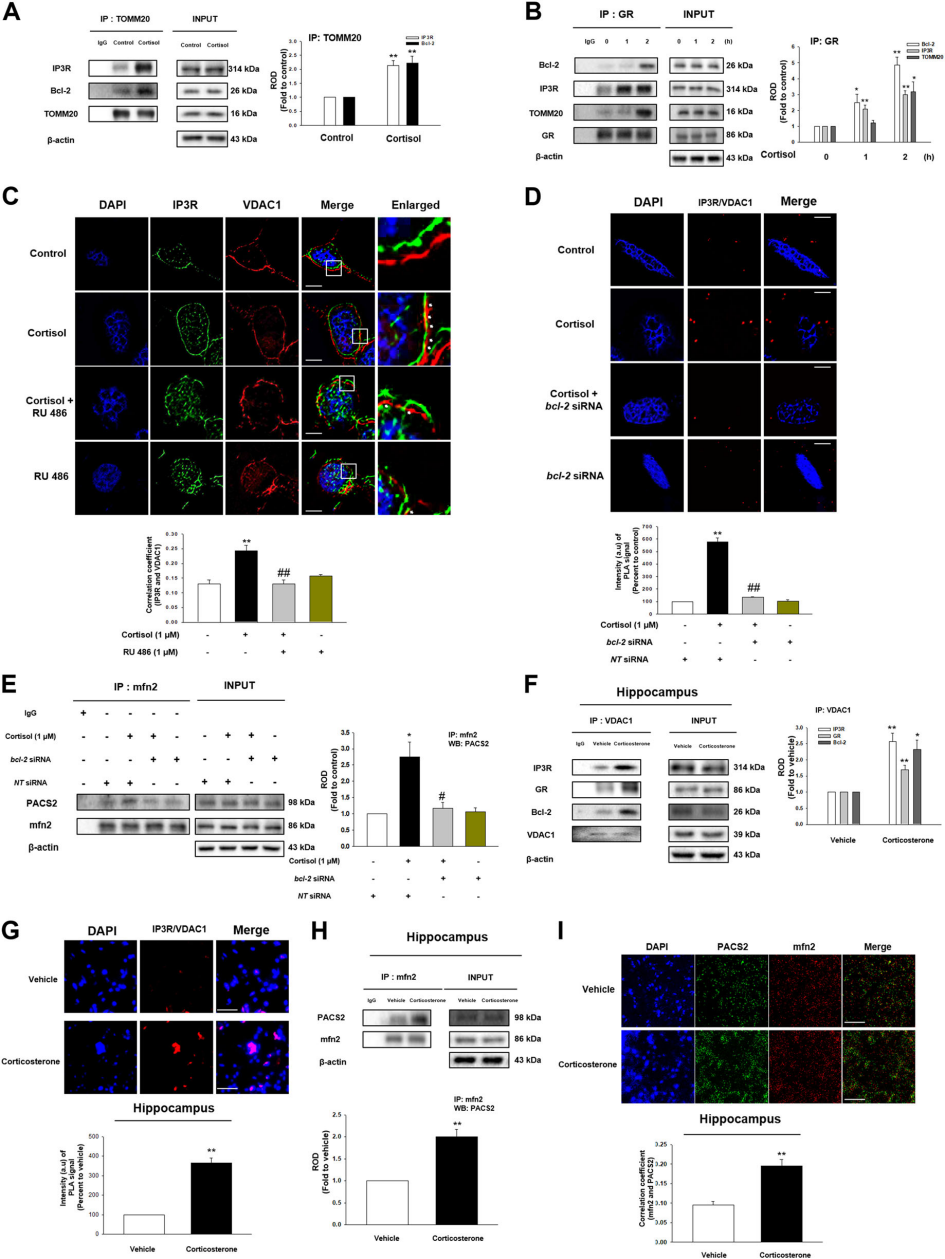
The effect of cortisol on ER-mitochondria contact via interaction between GR and Bcl-2. **a** The cells were incubated with cortisol (1 μM) for 2 h. TOMM20 was co-immunoprecipitated with anti-IP3R and -Bcl-2 antibodies (the left side). Expression of IP3R, Bcl-2, TOMM20, and β-actin in total cell lysates is shown in the right side. ^**^ indicates *p* < 0.01 vs. control. *n* = 4. **b** The cells were incubated with cortisol (1 μM) for various time. GR was co-immunoprecipitated with anti-Bcl-2, IP3R, and TOMM20 antibodies (the left side). Expression of Bcl-2, IP3R, TOMM20, GR, and β-actin in total cell lysates is shown in the right side. ^*, **^ indicates *p* < 0.05*, p* < 0.01 vs. control, respectively. *n* = 4. (**c**) The cells were incubated with RU 486 (1 μM) for 30 min before cortisol treatment (1 μM) for 2 h. Co-localization of IP3R (green) and VDAC1 (red) was visualized with SRRF imaging system. DAPI was used for nuclear counterstaining (blue). ^**^ indicates *p* < 0.01 vs. control and ^##^ indicates *p* < 0.01 vs. cortisol alone. Scale bars represent 20 μm (magnification, ×900). *n* = 5. **d** Knockdown of *bcl-2* was done using siRNA transfection for 24 h and then cells were treated with cortisol (1 μM) during 2 h. The cells underwent proximal ligation assay (PLA) and the red fluorescence indicates the co-localization between IP3R and VDAC1. DAPI was used for nuclear counterstaining (blue). Data are acquired by SRRF imaging system. ^**^ indicates *p* < 0.01 vs. control and ^##^ indicates *p* < 0.01 vs. cortisol alone. Scale bars represent 20 μm (magnification, ×900). *n* = 4. (**e**) Knockdown of *bcl-2* was done using siRNA transfection for 24 h and then cells were treated with cortisol (1 μM) during 2 h. mfn2 was co-immunoprecipitated with an anti-PACS2 antibody (the left side). Expression of PACS2, mfn2, and β-actin in total cell lysates is shown in the right side. ^*^ indicates *p* < 0.05 vs. control and ^#^ indicates *p* < 0.05 vs. cortisol alone. *n* = 4. **f** The hippocampus of mice exposed to vehicle or corticosterone (10 mg/kg) for 2 h was collected and lysed. VDAC1 was co-immunoprecipitated with anti-IP3R, GR, and Bcl-2 antibodies (the left side). Expression of IP3R, GR, Bcl-2, VDAC1, and β-actin in total cell lysates is shown in the right side. ^*^, ^**^ indicates *p* < 0.05*, p* < 0.01 vs. vehicle, respectively. *n* = 5. **g** Slide samples for IHC of mice with vehicle or corticosterone (10 mg/kg) for 2 h underwent PLA and the red fluorescence indicates the co-localization between IP3R and VDAC1. DAPI was used for nuclear counterstaining (blue). ^**^ indicates *p* < 0.01 vs. vehicle. Scale bars, 200 μm (magnification, ×200). *n* = 5. (**h**) The hippocampus of mice exposed to vehicle or corticosterone (10 mg/kg) for 2 h was collected and lysed. mfn2 was co-immunoprecipitated with an anti-PACS2 antibody (the left side). Expression of PACS2, mfn2, and β-actin in total cell lysates is shown in the right side. ^**^ indicates *p* < 0.01 vs. vehicle. *n* = 5. **i** Slide samples for IHC of mice with vehicle or corticosterone (10 mg/kg) for 2 h were immunostained with PACS2 (green) and mfn2 (red). DAPI was used for nuclear counterstaining (blue). Scale bars, 200 μm (magnification, ×200). ^**^ indicates *p* < 0.01 vs. vehicle. *n* = 5. All blot and immunofluorescence images are representative. Quantative data are presented as a mean ± S.E.M.

GR increases mitochondrial calcium by binding to Bcl-2, but underlying mechanism is not understood. One of major factors in facilitating the uptake of Ca^2+^ by mitochondria is the contact between ER and mitochondria where inositol 1,4,5-triphosphate receptor (IP3R) and voltage-dependent anion-selective channel 1 (VDAC1) bridge. Thus, we examined the effect of cortisol on ER-mitochondria coupling via GR binding to Bcl-2. We found the interaction between mitochondrial protein TOMM20 and mitochondria-associated membrane of ER (MAM) protein IP3R was increased with cortisol treatment, while the Bcl-2 was also translocated into mitochondria (Fig. 3a). We also showed that the binding between GR to Bcl-2, IP3R, and TOMM20 was increased with cortisol (Fig. 3b), suggesting that GR-Bcl-2 complex may increase ER-mitochondria contacts. As predicted, co-localization between IP3R and VDAC1 was strongly stimulated with cortisol treatment, but reduced by RU 486 (Fig. 3c). Proximal ligation assay (PLA) results also showed that cortisol triggered IP3R-VDAC1 interactions, which were decreased with the knockdown of *bcl-2* (Fig. 3d). We then assessed protein from which each organelle ligates to form ER-mitochondria coupling. Cortisol triggered the binding between mfn2 and phosphofurin acidic cluster sorting protein 2 (PACS2), reversed by knockdown of *bcl-2* (Fig. 3e). Meanwhile, this had no effect on vesicle-associated protein B (VAPB)-protein tyrosine phosphatase interacting protein 51 (PTPIP51) complex (Fig. S2A). Similar to in vitro results, corticosterone increased IP3R-VDAC1 interaction binding to GR and Bcl-2 in hippocampus of mice (Fig. 3f). Furthermore, we also found that ER-mitochondria contact was increased with corticosterone treatment in PLA results (Fig. 3g) via mfn2-PACS2 binding (Figs. 3h, i). Inhibiting ER-mitochondria tethering by knockdown of *bcl-2* or pretreatment of RU 486 also reduced A/T ratio, which indicates that this connectivity decreases microtubule stability (Figs. S2B – 2C).

### Involvement of ER-mitochondria connectivity in reduced ubiquitination of SCG10

**Fig. 4:**
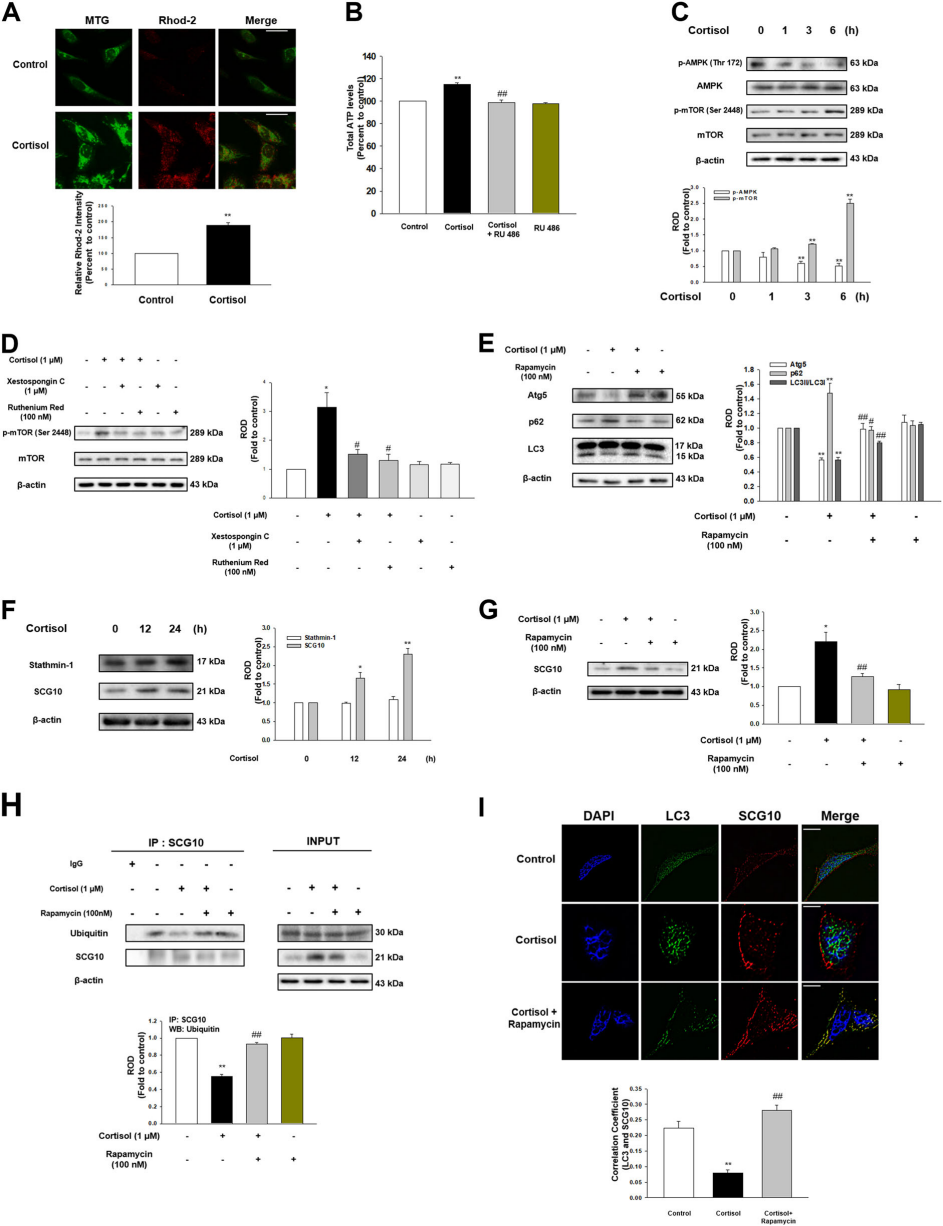
Cortisol inhibited selective autophagy towards SCG10 via activation of mTOR. **a** The cells were treated with cortisol (1 μM) for 3 h, and stained with rhod-2 (3 μM) for 1 h to detect mitochondrial Ca^2+^. After incubation, mitotracker green (MTG, 300 nM) was also stained to visualize mitochondria. The intensity of both rhod-2 (red) and MTG (green) was measured with Eclipse Ts2™ fluorescence microscopy. ^**^ indicates *p* < 0.01 vs. control. Scale bars represent 100 μm (magnification, ×400). *n* = 5. **b** The cells were treated with RU 486 (1 μM) for 30 min before cortisol (1 μM) for 6 h, and then reacted with ATP luciferase reagent. The ATP levels were detected with luminometer. ^**^ indicates *p* < 0.01 vs. control and ^##^ indicates *p* < 0.01 vs. cortisol alone. *n* = 6. **c** Time responses (0–6 h) of cortisol (1 μM) in phosphorylation of AMPK at Thr^172^ and mTOR at Ser^2448^ were shown. ^**^ indicates *p* < 0.01 vs. control. *n* = 4. **d** The cells were treated with xestospongin C (1 μM) for 2 h or ruthenium red (100 nM) for 30 min before cortisol (1 μM) for 6 h. p-mTOR (Ser^2448^), mTOR, and β-actin were detected in western blotting results. ^*^ indicates *p* < 0.05 vs. control and ^#^ indicates *p* < 0.05 vs. cortisol alone. *n* = 4. **e** The cells were pretreated with rapamycin (100 nM) for 30 min before cortisol (1 μM) for 24 h. Atg5, p62, LC3, and β-actin were detected with western blot. ^**^ indicates *p* < 0.01 vs. control. ^#^, ^##^ indicates *p* < 0.05*, p* < 0.01 vs. cortisol, respectively. *n* = 5. **f** Time responses (0–24 h) of cortisol (1 μM) in stathmin-1 and SCG10 expressions were shown. ^*^, ^**^ indicates *p* < 0.05*, p* < 0.01 vs. control, respectively. *n* = 5. **g** The cells were pretreated with rapamycin (100 nM) for 30 min before cortisol (1 μM) for 24 h. SCG10 and β-actin were detected with western blot. ^*^ indicates *p* < 0.05 vs. control and ^*##*^ indicates *p* < 0.01 vs. cortisol. *n* = 4. **h** The cells were pretreated with rapamycin (100 nM) for 30 min before cortisol (1 μM) for 24 h. SCG10 was co-immunoprecipitated with an anti-ubiquitin antibody (the left side). Expression of ubiquitin, SCG10, and β-actin in total cell lysates is shown in the right side. ^**^ indicates *p* < 0.01 vs. control and ^##^ indicates *p* < 0.01 vs. cortisol alone. *n* = 4. **i** The cells were pretreated with rapamycin (100 nM) for 30 min before cortisol (1 μM) for 24 h. Co-localization of LC3 (green) and SCG10 (red) was visualized with SRRF imaging system. DAPI was used for nuclear counterstaining (blue). ^**^ indicates *p* < 0.01 vs. control and ^##^ indicates *p* < 0.01 vs. cortisol alone. Scale bars represent 20 μm (magnification, ×1,000). *n* = 5. All blot and immunofluorescence images are representative. Quantative data are presented as a mean ± S.E.M.

ER-mitochondria contact by cortisol increased rhod-2 intensity which binds to mitochondrial Ca^2+^ (Fig. 4a). This indicates that Ca^2+^ was transferred through the IP3R-VDAC1 bridge, which mainly functions as a Ca^2+^ pathway from ER to mitochondria^11^. Induction of mitochondrial Ca^2+^ was decreased by the knockdown of *bcl-2* and pretreatment of xestospongin C (the potent membrane permeable IP3R antagonist) or VDAC1 inhibitor ruthenium red (Figs. S3A – 3C). Consistent Ca^2+^ transfer from ER to mitochondria is basically required to maintain ample NADH production^12^. We showed ATP production was increased approximately 20% with cortisol but reduced with RU 486 pretreatment (Fig. 4b). Increased ATP level generally leads to the deactivation of AMPK, which modulates the mTOR pathway. We showed that cortisol decreased AMPK activity, while stimulating mTOR phosphorylation (Fig. 4c). mTOR phosphorylation was remarkably reduced to the control level with xestospongin C or ruthenium red pretreatment, indicating that mitochondria calcium influx induced mTOR activation (Fig. 4d). mTOR inhibits autophagy function by directly phosphorylating or inhibiting ULK, AMBRA1, or Atg14^13^. Our results also showed that cortisol inhibited autophagy function represented by Atg5 reduction, increased p62 level, and decreased LC3B formation all of which were reversed by rapamycin (Fig. 4e). Autophagy is closely related to regulating protein level especially microtubule-associated proteins as cytoskeleton assembly plays a critical role in cell growth. Unlike mRNA expression of microtubule-associated proteins that remain unchanged (Fig. S3D), the expression of SCG10 was increased with cortisol (Fig. 4f) but decreased with rapamycin pretreatment (Fig. 4g), where cortisol had no effect on stathmin-1 expression. We therefore speculated that selective ubiquitination of SCG10 generally occurred, which was inhibited with cortisol. We showed ubiquitination of SCG10 was reduced with cortisol, but increased to the control level upon rapamycin pretreatment (Fig. 4h). Co-localization of LC3 and SCG10 was also promoted to the control level with pretreatment of rapamycin (Fig. 4i). We confirmed that increase in SCG10 level was dependent on the ubiquitination-proteasome system as proteasome inhibitor MG 132 decreased the SCG10 level whereas protein synthesis inhibitor cycloheximide had no effect on SCG10 expression (Fig. S3E). These results indicated that cortisol increased mitochondrial calcium influx which resulted in mTOR-dependent inhibition of autophagy and following SCG10 level increase.

### Glucocorticoid triggered microtubule dysfunction and kinesin-1 detachment leading to memory deficits through reducing trafficking of AMPAR and mitochondria into cell periphery

**Fig. 5:**
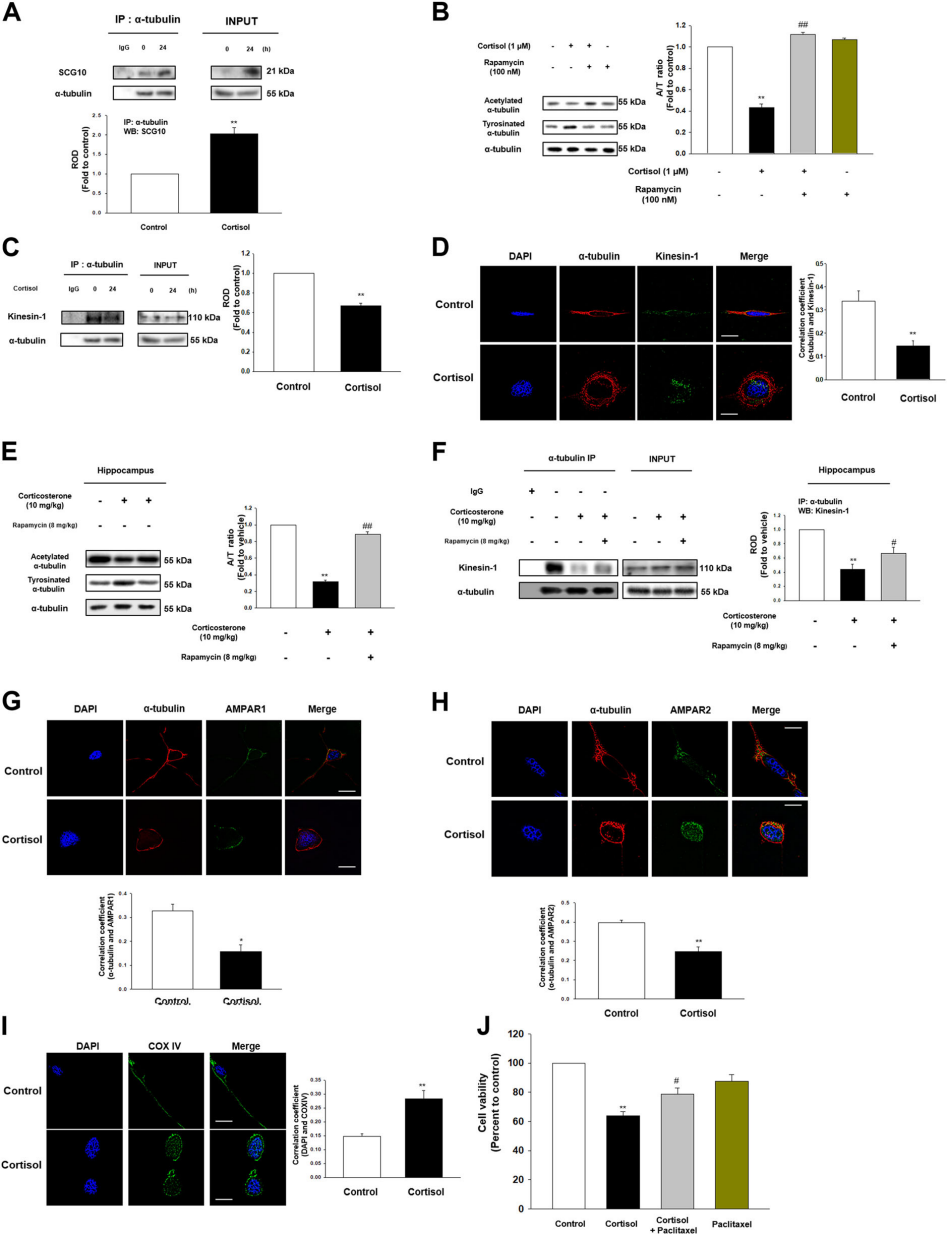
Glucocorticoid promoted microtubule destabilization and following transport impairment. **a** The cells were treated with cortisol (1 μM) for 24 h and lysed. α-tubulin was co-immunoprecipitated with an anti-SCG10 antibody (the left side). Expression of SCG10 and α-tubulin in total cell lysates is shown in the right side. ^**^ indicates *p* < 0.01 vs. control. *n* = 4. **b** The cells were pretreated with rapamycin (100 nM) for 30 min before cortisol (1 μM) for 48 h. Acetylated α-tubulin, tyrosinated α-tubulin, and α-tubulin were detected with western blot. ^**^ indicates *p* < 0.01 vs. control and ^##^ indicates *p* < 0.01 vs. cortisol alone. *n* = 4. **c** The cells were treated with cortisol (1 μM) for 24 h. α-tubulin was co-immunoprecipitated with an anti-kinesin-1 antibody (the left side). Expression of kinesin-1 and α-tubulin in total cell lysates is shown in the right side. ^**^ indicates *p* < 0.01 vs. control. *n* = 4. **d** The cells were incubated with cortisol (1 μM) for 24 h. Co-localization of α-tubulin (red) and kinesin-1 (green) was visualized with SRRF imaging system. DAPI was used for nuclear counterstaining (blue). ^**^ indicates *p* < 0.01 vs. control. Scale bars represent 20 μm (magnification, ×1000). *n* = 5. **e** The hippocampus of mice exposed to rapamycin (8 mg/kg) for 2 days before corticosterone (10 mg/kg) for 24 h was collected. Acetylated α-tubulin, tyrosinated α-tubulin, and α-tubulin were detected by western blot. ^**^ indicates *p* < 0.01 vs. vehicle. ^##^ indicates *p* < 0.01 vs. corticosterone alone. *n* = 5. **f** The hippocampus of mice exposed to rapamycin (8 mg/kg) for 2 days before corticosterone (10 mg/kg) for 24 h was collected and lysed. α-tubulin was co-immunoprecipitated with an anti-kinesin-1 antibody (the left side). Expression of kinesin-1 and α-tubulin in total cell lysates is shown in the right side. ^**^ indicates *p* < 0.01 vs. vehicle. ^#^ indicates *p* < 0.05 vs. corticosterone alone. *n* = 4. **g–h** The cells were incubated with cortisol (1 μM) for 48 h. Co-localization of α-tubulin (red) and AMPAR1/2 (green) was visualized with SRRF imaging system. DAPI was used for nuclear counterstaining (blue). ^*, **^ indicates *p* < 0.05*, p* < 0.01 vs. control, respectively. Scale bars represent 20 μm (magnification, ×1000). *n* = 5. **i** The cells treated with cortisol (1 μM) for 48 h were immunostained with DAPI (blue) and COX IV (green). ^**^ indicates *p* < 0.01 vs. control. Scale bars represent 20 μm (magnification, ×1000). *n* = 5. **j** The cells were pre-incubated with paclitaxel (10 μM) for 30 min before cortisol (1 μM) for 48 h. After treatment, water soluble tetrazolium salt (WST-1) assay was performed to measure cell viability. ^**^ indicates *p* < 0.01 vs. control and ^#^ indicates *p* < 0.05 vs. cortisol alone. *n* = 6. All blot and immunofluorescence images are representative. Quantative data are presented as a mean ± S.E.M.

Microtubule destabilization occurs when binding with SCG10 through activating/deactivating enzymes that regulate the PTM of α-tubulin. Increased SCG10-tubulin binding by cortisol was observed due to upregulation of SCG10 level (Fig. 5a). Thus, decreased A/T ratio of α-tubulins in cells upon cortisol was restored with pretreatment of rapamycin (Fig. 5b). Microtubule destabilization has been generally known to reduce the binding of kinesin-1, the motor protein that transports protein from cytosol close to the nucleus towards cell terminus. We showed decreased binding of kinesin-1 to α-tubulin with cortisol (Figs. 5c, d). Our animal studies also showed that corticosterone decreased A/T ratio in the hippocampus. On the other hand, rapamycin pretreatment not only restored A/T ratio (Fig. 5e), but also kinesin-1 binding to α-tubulin (Fig. 5f). As microtubule transports the important molecules into the necessary parts, we found that AMPAR1/2 did not closely interact with α-tubulin upon cortisol treatment (Figs. 5g, h). Mitochondria transport towards the cell terminus was also decreased by cortisol (Fig. 5i). Subsequently, cortisol reduced the cell viability, which was reversed by paclitaxel (Fig. 5j).

**Fig. 6:**
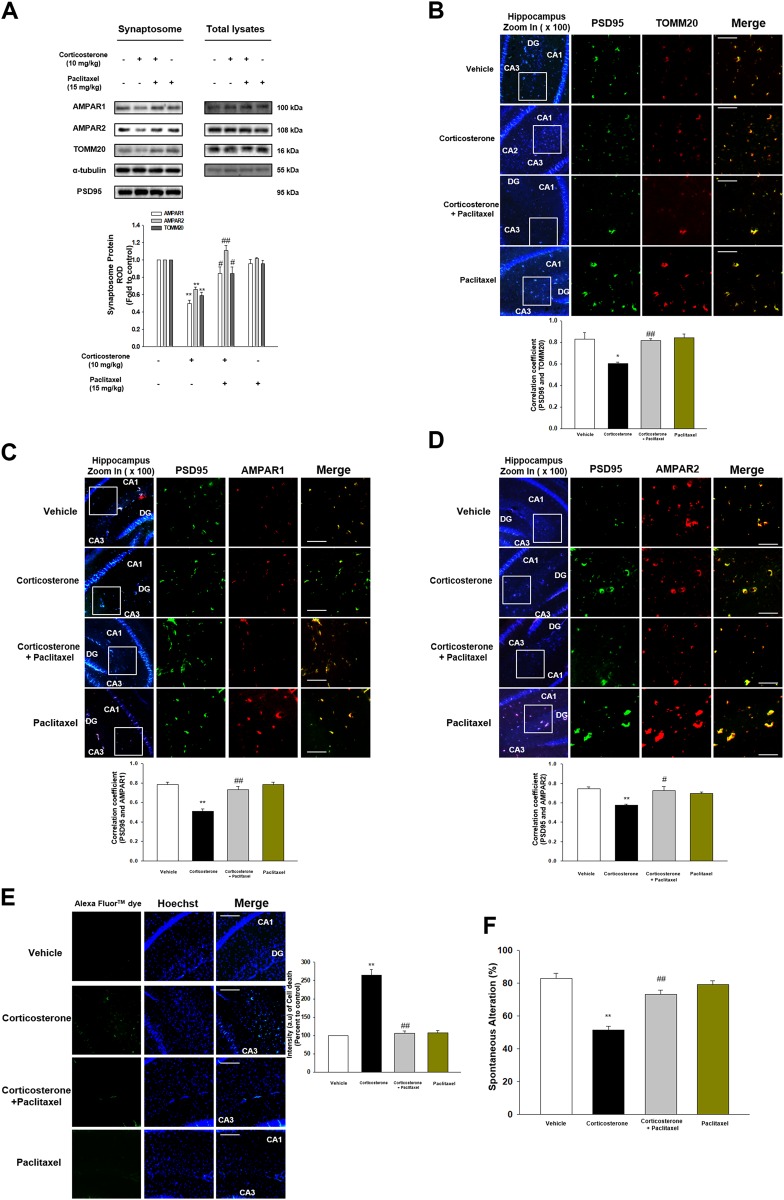
Corticosterone-induced memory impairment was attenuated by paclitaxel. **a** The synaptosome of hippocampus from mice exposed to vehicle, corticosterone (10 mg/kg), corticosterone with paclitaxel (15 mg/kg), and paclitaxel alone was isolated. AMPAR1, AMPAR2, TOMM20, α-tubulin, and PSD95 were detected. PSD95 was used as a loading control of synaptosome. ^**^ indicates *p* < 0.01 vs. vehicle. ^#^, ^##^ indicates *p* < 0.05, *p* < 0.01 vs. corticosterone treatment group, respectively. *n* = 6. **b–d** The mice were exposed to vehicle, corticosterone (10 mg/kg), corticosterone with paclitaxel (15 mg/kg), or paclitaxel. Slide samples for IHC were immunostained with PSD95 (green), TOMM20 or AMPAR1/2 (red), and DAPI (blue). ^*, **^ indicates *p* < 0.05, *p* < 0.01 vs. vehicle, respectively. ^#^, ^##^ indicates *p* < 0.05, *p* < 0.01 vs. corticosterone alone, respectively. Scale bars, 200 μm (magnification, ×200). *n* = 5. **e** TUNEL assay was performed using slide samples of hippocampus from mice with vehicle, corticosterone (10 mg/kg), corticosterone with paclitaxel (15 mg/kg), or paclitaxel. The intensity of green fluorescence indicates the amount of neuronal cell death. ^**^ indicates *p* < 0.01 vs. vehicle and ^##^ indicates *p* < 0.01 vs. corticosterone alone. Scale bars, 200 μm (magnification, ×200). *n* = 5. **f** The mice exposed to vehicle, corticosterone (10 mg/kg), corticosterone with paclitaxel (15 mg/kg), or paclitaxel were subjected to Y-maze test to evaluate memory function. ^**^ indicates *p* < 0.01 vs. vehicle and ^##^ indicates *p* < 0.01 vs. corticosterone alone. *n* = 6

We investigated whether microtubule stabilization reversed corticosterone effect that induced memory impairment and cell death in vivo. Corticosterone decreased translocation of AMPAR1/2 and TOMM20 into the synapse, which was recovered by paclitaxel injections (Fig. 6a). Similarly, corticosterone reduced co-localization between synaptic marker (post synaptic density 95-PSD95) and TOMM20 or AMPAR1/2 in hippocampal tissue, which was recovered with paclitaxel treatment (Figs. 6b–d). The TUNEL assay results showed that neuronal apoptosis was increased with corticosterone, which was reduced by paclitaxel treatment (Fig. 6e). In Y-maze test, mice with corticosterone showed memory impairment whereas the mice with paclitaxel pretreatment showed recovered cognition (Fig. 6f). Overall, our data supported that microtubule dysfunction led to failure of AMPAR or mitochondria trafficking into cell terminus, which promoted memory impairment and cell death.

## Discussion

**Fig. 7:**
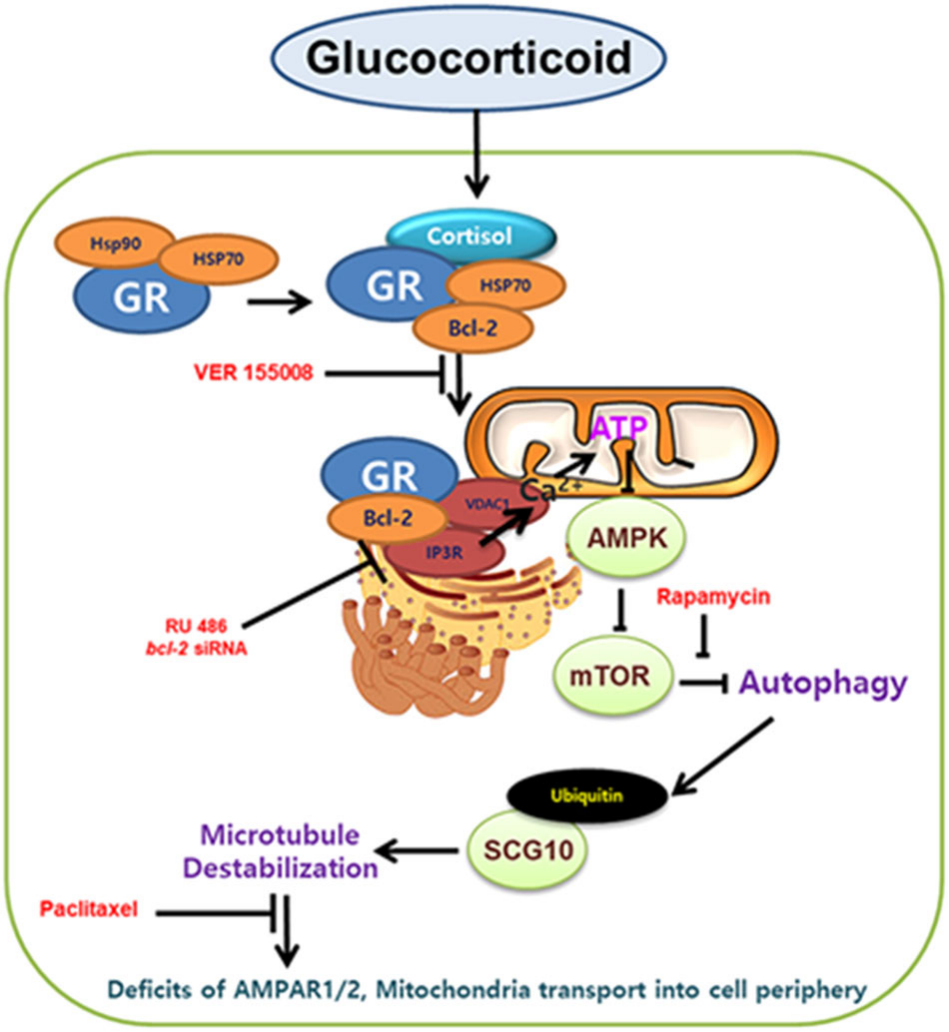
A hypothetical model for microtubule destabilization and memory deficits by the mitochondrial GR dependent action of glucocorticoid. GR bound to Hsp 70 is localized in mitochondria and forms complex with Bcl-2. GR-Bcl-2 complex promotes ER-mitochondria connection via IP3R-VDAC1 ligation and mitochondrial Ca^2+^ influx is induced. Subsequent mTOR activation inhibits selective autophagy which constantly triggers ubiquitination of SCG10. Increased SCG10 level evokes microtubule destabilization and trafficking of AMPAR or mitochondria into cell periphery is reduced due to kinesin-1 detachment. Thus, memory impairment and cell death follows, which is recovered by paclitaxel

Our study not only showed glucocorticoid changes microtubule dynamics through increasing ER-mitochondria coupling followed by reduction of SCG10 ubiquitination, but also demonstrated how microtubule dysfunction affects memory formation in both the animal model and SH-SY5Y cells (Fig. 7). We first demonstrated that microtubule destabilization in the hippocampus by corticosterone treatment eventually induced memory deficits. There are considerable research investigating how glucocorticoid induces microtubule dysfunction via amyloidosis and tau hyperphosphorylation by genomic pathway^5^ or membrane GR-dependent CREB pathway^14^, but the detailed mechanism elucidating the effect of glucocorticoid on microtubule has not been studied. Current research demonstrated, for the first time, that the effects of mitochondrial GR on the microtubule dysfunction and memory deficits. Our work identified Hsp70 played an important role in GR trafficking into mitochondria since Hsp70 is known to be mitochondrial chaperones importing various proteins into the mitochondria^15^. There are many debates that consider whether or not cortisol induced the translocation of GR into mitochondria. Chronic cortisol treatment triggered the reductions in GR trafficking into mitochondria due to the changes in GR expressions or PTM^16^. In contrast, cortisol usually stimulated GR movement into mitochondria with short term treatment representing an acute stress state, which is the same as our condition. Mitochondrial GR can directly affect the mitochondrial function including transcription of OXPHOS genes, ATP synthesis, and Ca^2+^ reuptake^17–19^. These changes in mitochondrial function alter microtubule dynamics since assembly or PTM of tubulin is mediated by mitochondrial Ca^2+^, which in turn affects ATP synthesis^16,20^. In particular, GR designated toward mitochondria has been widely accepted for binding to Bcl-2 to exert mitochondrial function change, but the detailed mechanism has not been implicated^21^. In this paper, we demonstrate how the GR-Bcl-2 complex increased the ER-mitochondria connectivity. Normally, about twenty percent of mitochondria are closely related to MAM. However, increased ER-mitochondria communication has been extensively observed in AD models, indicating that AD is deeply associated with the MAM function^22^. Interestingly, our works showed that ER-mitochondria contacts formed by GR-Bcl-2 complex triggered IP3R-VDAC1 bridging where both GR and Bcl-2 attach. Bcl-2 is previously described to bind to IP3R and mediate Ca^2+^ transfer from ER into mitochondria^11,23,24^. Surprisingly, we also observed GR interacted with IP3R, which was not observed in previous research. Indeed, we could assume that GR-Bcl-2 complex binds to the MAM region serving as scaffold to help ER-mitochondria coupling, although the exact mechanism needs further investigation. Several tethering proteins function to increase interaction between MAM and mitochondria such as PACS2-mfn2 and VAPB-PTPIP51^25^. In our work, cortisol formed mfn2-PACS2 interaction, one of the major tethering protein complexes stabilizing the ER-mitochondria network, which was decreased with knockdown of *bcl-2*^11^. We also confirmed that inhibiting ER-mitochondria coupling restored A/T ratio, suggesting that GR translocation into mitochondria is highly associated with microtubule destabilization through integration of ER and mitochondria.

Recent studies have concentrated on elucidating new therapeutic approach to AD through an in-depth inspection on the downstream effect of ER-mitochondria interaction since it precedes the accumulation of plaques or tangles^22,26^. We showed that IP3R-VDAC1 bridging increased mitochondrial Ca^2+^ which finally culminated in autophagy dysfunction^12,23,27^. Ca^2+^ overload in mitochondria conversely leads to increased ATP synthesis via stimulating OXPHOS enzymes^28^. Although some previous reports suggested that chronic glucocorticoid treatment reduced ATP synthesis due to prolonged mitochondria damage and different tendency of regulating OXPHOS genes, many studies showed glucocorticoid promotes ATP synthesis by increasing mitochondrial calcium storing capacity or GR binding to the promoters of OXPHOS genes in mitochondrial DNA^17,18^. With increased ATP level, the deactivation of AMPK either phosphorylates TSC2 or raptor to inhibit the autophagy process^13^. Several studies showed that glucocorticoid elevated autophagy-related genes stimulating autophagy process in lymphocytes and osteoblasts^29,30^. However, the glucocorticoid dosage to treat such inflammatory diseases in other types of tissues rather than brain is different from the stress-induced glucocorticoid level since it is designed to efficiently induce autophagic cell death. In contrast, our results showed that the deactivation of AMPK triggered phosphorylation of its substrate mTOR leading to defective autophagy, which agrees with glucocorticoid effect on altered mTOR signaling of brain in the previous report^31^. Our works showed that glucocorticoid inhibited selective autophagy towards the SCG10. Selective autophagy via ubiquitination is important to avoid the accumulation of redundant proteins^32^. In particular, selective autophagy serve as crucial regulator of microtubules responsible for migration, development, and differentiation^33,34^. There are many proteins subject to selective autophagy inducing axonal degeneration such as α-synuclein, huntingtin proteins, stathmin, and neurofilament. Namely, if defective autophagy clearance occurs, the microtubule dysfunction is likely to follow. Previous reports suggest that autophagy induction stabilizes neuronal microtubule via decreasing SCG10, indicating that autophagy plays a pivotal role in regulating SCG10 level^35,36^. Our results also indicated that selective ubiquitination of SCG10 among stathmin family was reduced by cortisol whereas inhibition of mTOR eventually triggered reduction in SCG10 level. However, the regulatory mechanism of SCG10 ubiquitination in neuronal cells should be further investigated.

SCG10-dependent changes in microtubule dynamics were already reported to be involved in memory formation, but the exact mechanism of how glucocorticoid induces memory deficits via microtubule dysfunction is far from clear. Mounting evidence also suggest that multiple bindings between kinesin-1 and α-tubulin move necessary components further than single binding, indicating that the kinesin-1 detachment from α-tubulin can dramatically fail to transfer cargos into cell terminus^37^. Thus, microtubule destabilization due to increased tyrosination of α-tubulin triggers reduction in the binding of kinesin-1 motor protein to α-tubulin^9^. Accordingly, we exhibited that decreasing SCG10 level with rapamycin restored the A/T ratio and kinesin-1 binding to α-tubulin. Stathmin-dependent changes in microtubule stability are widely known to influence memory formation. Several reports showed that transiently unstable microtubule is necessary for memory consolidation^9^. However, this report focused on the phosphorylation state of stathmin which inactivates its depolymerizing. On the other hand, previous research reported that excessive SCG10-tubulin binding significantly affects the microtubule stability, resulting in decreased intracellular transport into the synaptic sites^9^. Given the observations, we speculated that the glucocorticoid-treated cells are likely to fail transporting the necessary components for memory formation towards the cell periphery. Microtubule transports AMPARs and mitochondria to establish the memory consolidation and synaptic strength^38^ since they play a major role in establishing hippocampal LTP^24,39^. For example, the stathmin family regulates binding between AMPAR2 and kinesin-1 in synaptic sites maintaining memory function^40^. However, the relationship between glucocorticoid and trafficking of AMPAR or mitochondria has not yet been studied. We identified in vitro and in vivo studies demonstrated that the AMPAR and mitochondria transport into cellular extremities and synapse was significantly reduced upon glucocorticoid treatment. Furthermore, these works agree with the previous report demonstrating that excessive glucocorticoid triggers the synaptic dysfunction and hippocampal apoptosis with increased caspases^41^. We then used microtubule-stabilizing drug for helping microtubule become acetylated to confirm that microtubule is essential for intracellular transport^42^. Using paclitaxel that stimulates the recovery of the AMPAR and mitochondria levels in cell periphery, memory formation was restored to normal functions and the neuronal cell death was reduced, indicating that microtubule dynamics play an important role in memory function via normalizing intracellular transport. Therefore, our data demonstrate that glucocorticoid impairs the localization of memory-related receptors and mitochondria in the synapse by SCG10-mediated changes in microtubule stability.

In conclusion, the results of this study showed that glucocorticoid impairs microtubule function by reorganizing the ER-mitochondria interaction which inhibits selective autophagosome formation of SCG10 through inducing mitochondrial calcium influx and subsequent activating mTOR pathway. Furthermore, we also demonstrated the underlying mechanisms that microtubule destabilization by glucocorticoid finally decreases memory formation and induces neurodegeneration via inhibiting the transport of AMPAR and mitochondria into the cell terminus. Thus, our approach to investigate the new signaling pathways of glucocorticoid on memory deficits via microtubule dysfunction in the animal model and neuroblastoma cells can provide potential therapeutic targets that control the memory recovery.

## Materials and methods

### Materials

Cells from the human neuroblastoma cell line SH-SY5Y were obtained by Korean Cell Line Bank (Seoul, Korea). Fetal bovine serum (FBS) and serum replacement (SR) were purchased from Hyclone (Logan, UT, USA) and Gibco (Grand Island, NY, USA), respectively. The antibodies of mfn2 (sc-515647), VDAC1 (sc-390996), stathmin-1 (sc-48362), SCG10 (sc-135620), Bcl-2 (sc-492), p-mTOR (Ser^2448^, sc-8319), p-AMPK (Thr^172^, sc-33524), VAPB (sc-293364), and β-actin (sc-47778) were acquired from Santa Cruz Biotechnology (Santa Cruz, CA, USA). The antibodies of TOMM20 (ab56783), IP3R (ab5804), and the product of xestospongin C were purchased from Abcam (Cambridge, England). Horseradish peroxidase (HRP)-conjugated goat anti-rabbit IgG was obtained from Jackson Immunoresearch (West Groove, PA, USA). The antibodies of AMPAR1 (AB1504), AMPAR2 (AB1768-I), and PSD95 (MAB1596) were purchased from EMD Millipore (Billerica, MA, USA). The antibodies of AMPK (2532 S), mTOR (2983 S), and GR (12041 S) were purchased from Cell Signaling Technology, Inc. (Danvers, MA, USA). The antibodies of LC3 (NB100-2220), p62 (NBP1-48320), Atg5 (NB110-53818), and kinesin-1 (NBP2-21667) were purchased from Novus Biologicals (Littleton, CO, USA). The antibodies of ubiquitin (701339) and PSD95 (51-6900) were obtained from Thermo Fisher (Rockford, IL, USA). The antibodies of COX IV (CSB-PA001765), Hsp90 (CSB0PA005496), Hsp70 (CSB-PA863082HA01HU), PACS2 (CSB-PA017372LA01HU), PTPIP51 (CSB-PA836307DSR2HU), and the chemical cortisol-BSA were obtained from Cusabio (Wuhan, Hubei, China). Cortisol, corticosterone, BSA, RU 486, paclitaxel, DAPI, actinomycin D, rapamycin, cycloheximide, MG132, Hoechst, ruthenium red, and the antibodies of acetylated α-tubulin (T7451), tyrosinated α-tubulin (T9028), α-tubulin (T6074) were purchased from Sigma Chemical Company (St. Louis, MO, USA). VER 155008 was obtained from Calbiochem (Merk Millipore, Darmstadt, Germany).

### Cell culture

The SH-SY5Y cells were cultured in high glucose Dulbecco’s Modified Eagle Medium (DMEM, Hyclone) containing 10% FBS and 1% antibiotic-antimycotic mixture. Cells were cultured in 60, 100 mm diameter culture dishes, or a 96-well plate in an incubator kept at 37 °C with 5% CO_2_. Cells were incubated for 72 h and then the medium was replaced with serum-free DMEM containing 1% SR and 1% antibiotic-antimycotic solution for 24 h. The in vitro studies using SH-SY5Y cells are designed to investigate the effect of cortisol on microtubule dynamics-dependent intracellular trafficking.

### Experimental design of animal study

Male ICR mice exposed to corticosterone mimic the stress-induced mouse model since corticosterone is primarily responsive steroid hormone to stress. The hippocampus of mice was mainly used for evaluating glucocorticoid effect on microtubule dynamics since the hippocampus has the most abundant GRs among the brain. Male ICR mice aged 7 weeks were used, in compliance and approval with the Institutional Animal Care and Use Committee of Seoul National University (SNU-171017-9). Animals were housed 6 per cage under standard environmental conditions (22 °C relative humidity 70%; 12 h light: dark cycle; *ad libitum* access to food and drinking solution). Total seventy two of 7-week-old male ICR mice were used for the in vivo study. Six mice were utilized for each group throughout the study. Applying size of samples (minimum of *n* = 3) can be acceptable if very low *p* values are observed rather than the large size of N including interfering results^43^. Therefore, we set the minimum of *n* = 4 (western blotting, Immunohistochemistry (IHC)) and *n* = 6 (behavior test) to gain statistical powers according to the previous published article of *brain*^44^. The experiments were designed in compliance with the ARRIVE guidelines. Allocations of animals were randomly done to minimize the effects of subjective bias. No exclusion of data obtained from samples was done.

Corticosterone (10 mg/kg) was dissolved in the solution containing 50% propylene glycol in PBS and injected intraperitoneally^14^. Vehicle-treated mice were similarly injected with the solution containing propylene and PBS. Rapamycin (8 mg/kg) were dissolved in the solution containing 1% DMSO and 99% corn oil (Sigma Chemical Company). The dosage and treatment period of rapamycin is modified from previous reports^45^. The vehicle solution includes the 1% DMSO and 50% propylene glycol in PBS. Paclitaxel (15 mg/kg) was dissolved in the solution containing 50% β-cyclodextrin and treated 3 h before the corticosterone treatment following the modified method and dosage of previous report^46^. Vehicle-treated mice were similarly injected with the solution containing propylene glycol and β-cyclodextrin. Mice were monitored twice a day during all experiments.

### Y-maze spontaneous alteration test

Y-maze spontaneous alteration test is based on the innate willingness of rodents to differently explore new environments. This behavior test is frequently used for quantifying the cognitive deficits of the animals. Rodents usually prefer to challenge a new arm of the Y-maze rather than returning back to the one which was previously visited. Before the test, the animals were placed in the home cage at the testing room for 3 h to minimize the effects of stress on behavior. The mice were placed in the Y-shaped maze purchased from Sam-Jung Company (Seoul, Korea). The mice were allowed to explore the open field for 10 min, and at the same time, the number of arm entries and triads were recorded to calculate the percentage of alteration. Only an entry when all four limbs were within the arm was counted. The alteration amount represents the number of alterations which was divided by total triads (total entries-2). When the animals show higher alteration percentage, the animals tend to maintain the memory function.

### Immunohistochemistry (IHC)

Mice underwent deep anesthesia with zoletil (50 mg/kg) and were perfused transcardially with calcium-free Tyrode’s solution followed by a fixative including 4% paraformaldehyde in 0.1 M phosphatebuffer (pH 7.4). The removed brain was post-fixed for 2 h, and subsequently placed in 30% sucrose in PBS for 24 h at 4 °C. Serial transverse sections (40 μm) were performed using a cryostat (Leica Biosystems, Nussloch, Germany). The brain tissues containing hippocampus were fixed with 4% paraformaldehyde and then pre-blocked with 5% normal goat serum (Sigma Chemical Company) containing 0.1% Triton X-100 in PBS at room temperature for 1 h. Samples were incubated with primary antibodies overnight at 4 °C, followed by secondary antibodies for 2 h at room temperature. Completed samples were visualized by using Eclipse Ts2™ fluorescence microscopy (Nikon, Tokyo, Japan). The fluorescent intensity analysis was undertaken using Fiji software.

### Synaptic protein extraction

Synaptosome of hippocampus was extracted using Syn-Per synaptic protein extraction reagent (Thermo Fisher). Hippocampus was homogenized with Dounce grinder with 20 slow strokes. The homogenates underwent centrifugation at 1200×*g* for 10 min at 4 °C. After discarding the pellet, the supernatant was centrifuged at 15,000×*g* for 20 min at 4 °C. The supernatant part is cytosolic fraction and the pellet (synaptosome) was dissolved with the extraction reagent.

### TUNEL assay

The cryo-sectioned brain tissue underwent the TUNEL assay using Click-iT^TM^ TUNEL Alexa Fluor^TM^ 488 Imaging Assay (Thermo Fisher) to evaluate the apoptosis of hippocampal neuronal cells. The samples were fixed with 4% paraformaldehyde for 15 min and then placed with 0.25% triton X-100 in PBS for 20 min at room temperature. The processes of TdT incorporation of EdUTP into dsDNA strand breaks and incubation with fluorescent dye detecting the EdUTP were conducted according to the manufacturer’s instructions. Images were obtained by Eclipse Ts2™ fluorescence microscopy. The fluorescent intensity analysis was performed using Fiji software.

### Reverse transcription-polymerase chain reaction (RT-PCR) and real-time PCR

The SH-SY5Y cells were treated for 12 h with cortisol, and the total RNA was extracted using MiniBEST Universal RNA Extraction Kit (TaKaRa, Otsu, Shinga, Japan). Reverse transcription was performed using 1 μg of RNA with a Maxime RT-PCR PreMix Kit (Intron Biotechnology, Seongnam, Korea) to obtain cDNA. Two microliters of cDNA was then amplified using Quanti NOVA SYBR Green PCR Kits (Qiagen, Hilden, Germany). Real-time quantification of RNA targets was performed in a Rotor-Gene 6000 real-time thermal cycling system (Corbett Research, NSW, Australia). The primers for microtubule associated proteins were purchased from the Bioneer Corporation (Daejeon, Korea). The reaction mixture (20 μl) contained 200 ng of total RNA, 0.5 mM of each primer, and appropriate amounts of enzymes and fluorescent dyes as recommended by the manufacturer. The real-time PCR was performed as follows: 15 min at 95 °C for DNA polymerase activation; 15 s at 95 °C for denaturing; and 50 cycles of 15 s at 94 °C, 30 s at 54 °C, and 30 s at 72 °C. Data were collected during the extension step, and analysis was performed using the software provided; melting curve analysis was performed to verify the specificity and identity of the PCR products. Normalization of gene expression levels was performed by using the *β-actin* gene as a control.

### Western blot analysis

Harvested tissues or cells were incubated with the appropriate buffer for 30 min on ice. The lysates were cleared by centrifugation (10,000×*g* at 4 °C for 30 min) and the supernatant was collected. To evaluate the protein concentration, the bicinchoninic acid (BCA) assay kit (Bio-Rad, Hercules, CA, USA) was used. Equal amounts of sample proteins (1–5 μg) were prepared for 8-15% SDS-PAGE and then transferred to a polyvinylidene fluoride membrane. Subsequently, the membranes were blocked with 5% BSA or 5% skim milk (Gibco) in TBST solution for 30 min. Blocked membranes were incubated with primary antibody overnight at 4 °C. The membranes were then washed and incubated with the HRP-conjugated secondary antibody at room temperature for 2 h. The western blotting bands were visualized by using chemiluminescence solution (Bio-Rad) and the densitometry analysis for quantification was carried out by using Image J software.

### Small interfering RNA (siRNA) transfection

Cells were grown until 60% of the surface of the plate. Prior to cortisol treatment, siRNAs specific for *bcl-2* and *nontargeting* (*NT*) obtained from Bioneer Corporation (Daejeon, Korea), and Dharmacon (Lafayette, CO, USA), respectively, were transfected to cells for 24 h with turbofect transfection reagent (Thermo Fisher) according to the manufacturer’s instructions. The concentration of each transfected siRNA was 25 nM. *NT* siRNA was used as the negative control.

### Immunocytochemistry

Cells on a confocal dish (Thermo Fisher) were fixed with 80% acetone for 10 min. Then, cells were incubated with 5% normal goat serum in PBS and incubated with primary antibody for overnight in 4 °C. Next, the cells were incubated for 2 h at room temperature with Alexa fluor secondary antibody. Images were obtained by Eclipse Ts2™ fluorescence microscopy or super-resolution radial fluctuations (SRRF) imaging system (Andor Technology, Belfast, UK)^47^. The fluorescent intensity analysis and co-localization analysis with Pearson’s correlation coefficient were acquired by Fiji software.

### Measurement of cellular ATP levels

Intracellular ATP concentration level of cells was measured using ATP Bioluminescent HSII kit (Roche, Basel, Switzerland) according to the manufacturer’s instructions. ATP levels were detected with luminometer (Victor3; Beckman Coulter, Fullerton, CA, USA) and normalized to total protein concentration.

### Co-immunoprecipitation

The magnetic bead conjugated with specific primary antibodies was immobilized according to the supplier’s instructions. The total lysates of cells (300 μg) was incubated with 10 μg of primary antibody for overnight at 4 °C. Magnetic beads were spun-down by magnet and then collected. The antibody-bound protein was acquired by incubation in elution buffer (Thermo Fisher).

### In situ proximal ligation assay (PLA)

Duolink™ in situ PLA was performed according to the manufacturer’s instructions (Sigma Chemical Company). After fixation and blocking at 37 °C, primary antibodies against rabbit anti-IP3R and mouse anti-VDAC1 were diluted in antibody diluent and then incubated overnight at 4 °C. The Duolink™ secondary antibodies against the particular primary antibodies were applied for 1 h at 37 °C. Ligase was then added for 1 h at 37 °C and then amplification was done. Fluorescent images were visualized with Eclipse Ts2™ fluorescence microscopy or SRRF imaging system.

### Water soluble tetrazolium salt (WST-1) assay

WST-1 assay was used for determining the cell proliferation and viability in vitro model. After treatment, cells were incubated in 10 μl of EZ-Cytox^TM^ solution including WST-1 in 100 μl of medium for 30 min at 37 °C with 5% CO_2_. The absorbance of each sample using a microplate reader was measured at a wavelength of 450 nm.

### Statistical analysis

Results are expressed as mean value ± standard error of mean (S.E.M.) and analyzed with the sigma plot 10 software. All experiments were analyzed by ANOVA, and some experiments which needed to compare with 3 groups were examined by comparing the treatment means to the control using a Bonferroni-Dunn test. A result with a *p* value of <0.05 was considered statistically significant.

## Electronic supplementary material

Supplementary figures
