# Supplementary material for: Glucocorticoid-mediated ER-mitochondria contacts reduce AMPA receptor and mitochondria trafficking into cell terminus via microtubule destabilization
Source: Cell Death Dis. 2018 Nov 14;9(11):1137. doi: 10.1038/s41419-018-1172-y (PMC6235892; doi:10.1038/s41419-018-1172-y)
Supplement: Supplementary file 1 — Supplementary figures [file 41419_2018_1172_MOESM1_ESM.docx]

**Supplementary Materials**

**Figure S1**

**
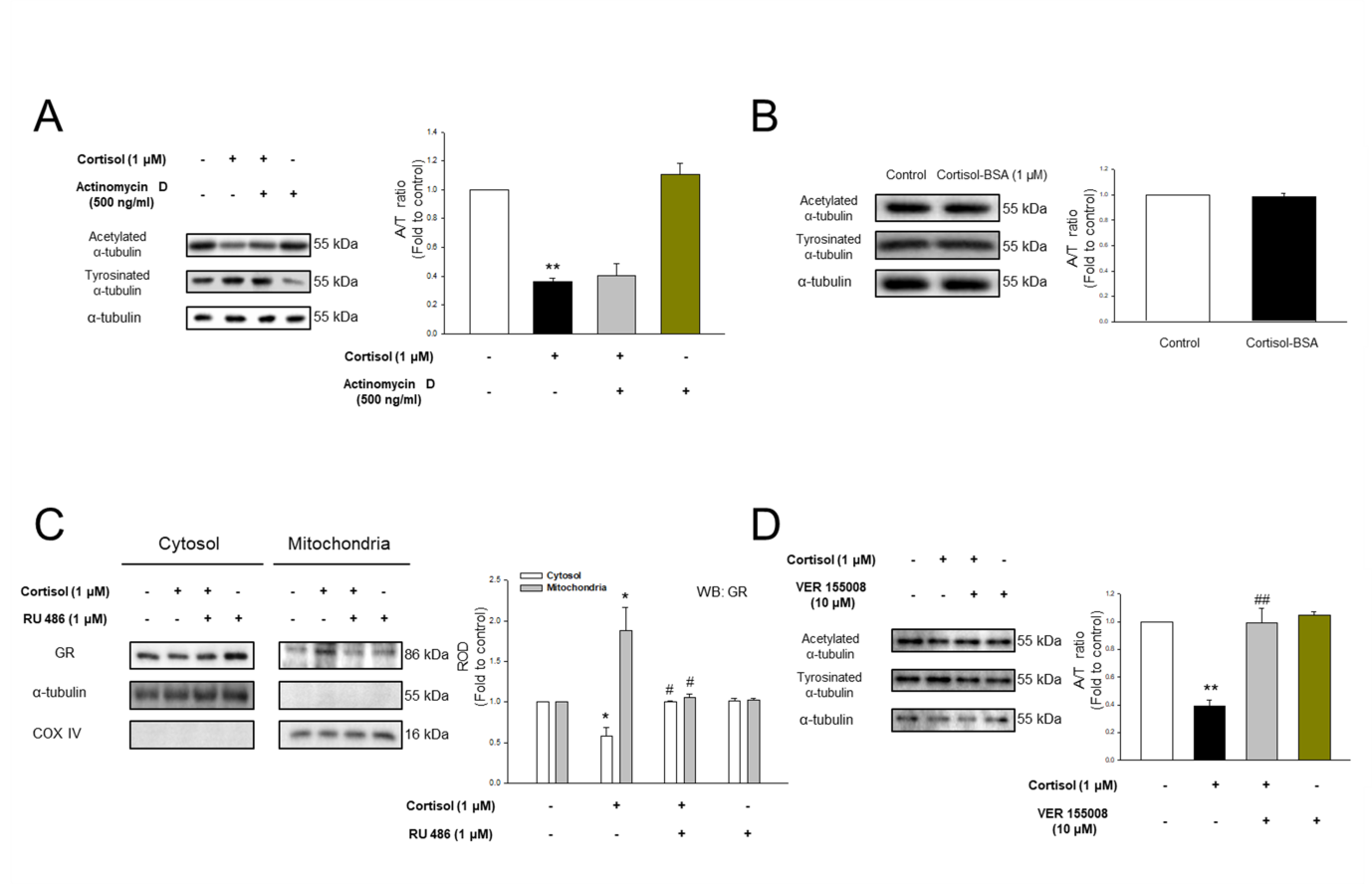
**

**Figure S1. Microtubule dysfunction by cortisol is mediated by mitochondrial GR in SH-SY5Y cells.** (**A**) The cells were incubated with actinomycin D (500 ng/ml) for 30 min before cortisol treatment (1 μM) for 48 h. Acetylated α-tubulin, tyrosinated α-tubulin, and α-tubulin were detected. Data are reported as a mean ± SE of four independent experiments. *^**^* indicates *p<0.01* versus control. (**B**) The cells were treated with cortisol-BSA (1 μM) for 48 h. Acetylated α-tubulin, tyrosinated α-tubulin, and α-tubulin were detected. n=4. (**C**) The cells were incubated with RU 486 (1 μM) for 30 min before cortisol treatment (1 μM) for 2 h. GR, α-tubulin, and COX IV were detected. Cytosolic and mitochondrial protein expressions were normalized by α-tubulin and COX IV, respectively, in western blotting results. Data are reported as a mean ± SE of four independent experiments. *^*^* indicates *p<0.05* versus control and *^#^* indicates *p<0.05* versus cortisol. (**D**) The cells were incubated with VER 155008 (10 μM) for 30 min before cortisol treatment (1 μM) for 48 h. Acetylated α-tubulin, tyrosinated α-tubulin, and α-tubulin were detected. Data are reported as a mean ± SE of four independent experiments. *^**^* indicates *p<0.01* versus control and *^##^* indicates *p<0.01* versus cortisol.

**Figure S2**

**
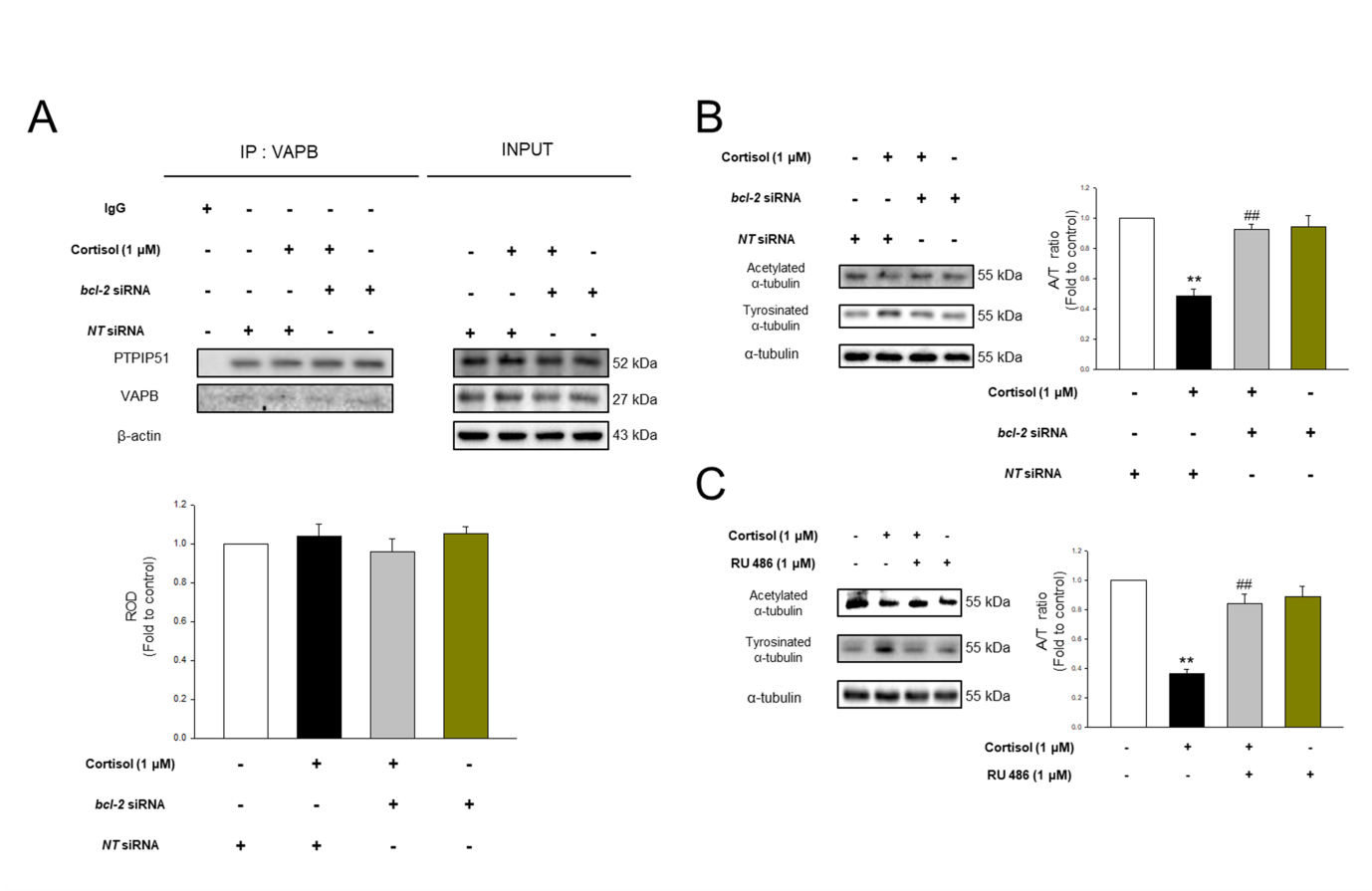
**

**Figure S2. Increased ER-mitochondria contact by cortisol induced microtubule destabilization.**

(**A**) Knockdown of *bcl-2* was done using siRNA transfection for 24 h and then cells were treated with cortisol (1 μM) during 2 h. VAPB was co-immunoprecipitated with an anti-PTPIP51 antibody (the left side). Expression of PTPIP51, VAPB, and β-actin in total cell lysates is shown in the right side. n=4. (**B**) Knockdown of *bcl-2* was done using siRNA transfection for 24 h and then cells were treated with cortisol (1 μM) during 48 h. Acetylated α-tubulin, tyrosinated α-tubulin, and α-tubulin were detected. Data are reported as a mean ± SE of four independent experiments. *^**^* indicates *p<0.01* versus control and *^##^* indicates *p<0.01* versus cortisol. (**C**) The cells were incubated with RU 486 (1 μM) for 30 min before cortisol treatment (1 μM) for 48 h. Acetylated α-tubulin, tyrosinated α-tubulin, and α-tubulin were detected. Data are reported as a mean ± SE of four independent experiments. *^**^* indicates *p<0.01* versus control and *^##^* indicates *p<0.01* versus cortisol.

**Figure S3**

**
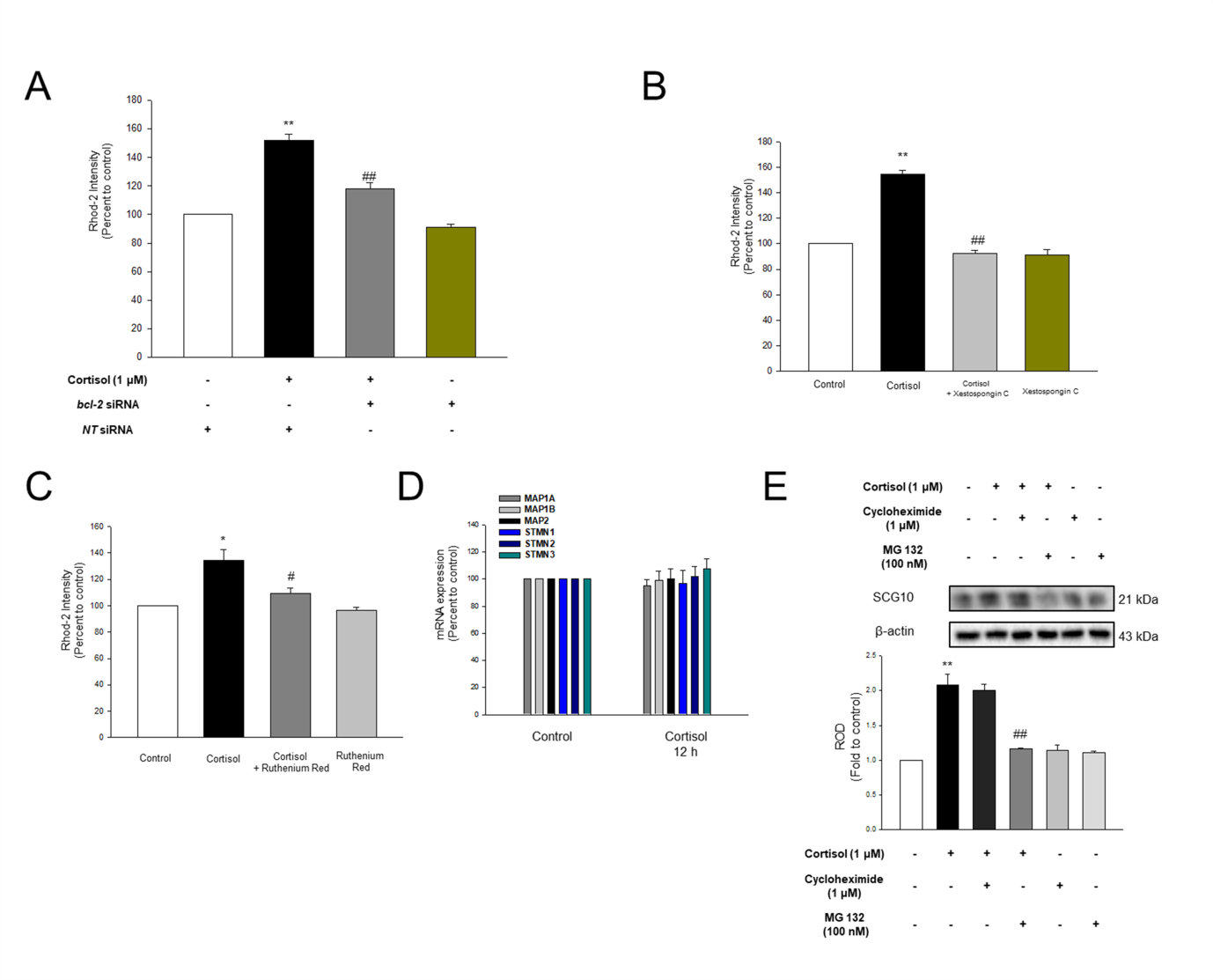
**

**Figure S3. Mitochondria Ca^2+^ induced by ER-mitochondria connectivity increased SCG10 level.** (**A**) Knockdown of *bcl-2* was done using siRNA transfection for 24 h and then cells were treated with cortisol (1 μM) during 3 h. Then the cells were stained with rhod-2 (3 μM) for 1 h to detect mitochondrial Ca^2+^ by luminometer. Data are reported as a mean ± SE of six independent experiments. *^**^* indicates *p<0.01* versus control and *^##^* indicates *p<0.01* versus cortisol. (**B**) The cells were treated with xestospongin C (1 μM) for 2 h before cortisol (1 μM) for 3 h. Then the cells were stained with rhod-2 (3 μM) for 1 h to detect mitochondrial Ca^2+^ by luminometer. Data are reported as a mean ± SE of six independent experiments. *^**^* indicates *p<0.01* versus control and *^##^* indicates *p<0.01* versus cortisol. (**C**) The cells were treated with ruthenium red (100 nM) for 30 min before cortisol (1 μM) for 3 h. Then the cells were stained with rhod-2 (3 μM) for 1 h to detect mitochondrial Ca^2+^ by luminometer. Data are reported as a mean ± SE of six independent experiments. *^*^* indicates *p<0.05* versus control and *^#^* indicates *p<0.05* versus cortisol. (**D**) The cells were treated with cortisol (1 μM) during 12 h, and mRNA was extracted. Real time PCR was performed to measure mRNA expressions. β-actin was used for loading control. n=6. (**E**) The cells were treated with cycloheximide C (1 μM) or MG 132 (100 nM) for 30 min before cortisol (1 μM) for 24 h. SCG10 and β-actin were detected in western blotting results. Data are reported as a mean ± SE of four independent experiments. . *^**^* indicates *p<0.01* versus control and *^##^* indicates *p<0.01* versus cortisol.
